# Frequent genetic defects in long-term survivors for more than 26 years in the absence of antiretroviral therapy in Korea: its association with ginseng treatment

**DOI:** 10.1186/1742-4690-10-S1-P14

**Published:** 2013-09-19

**Authors:** Young-Keol Cho, Ba-Reum Kim, Jung-Eun Kim

**Affiliations:** 1 Microbiology, University of Ulsan College of Medicine, SEOUL, Republic of Korea

## Background

The association between long-term nonprogressors (LTNPs) and genetic defects in HIV-1 is not clear because of paucity of genetic defects in LTNPs. To date, there is only a report on gross deletion in 2 genes of HIV-1. Ginseng has been used as the premier medicinal plant for a thousand years [1]. Korean red ginseng (KRG) has been applied to HIV-1 infected patients since November 1991 [2]. We have found that most HIV-1 infected patients treated with KRG for a significant period slowly progressed to AIDS and revealed a high frequency of genetic defects.

## Materials and methods

A total of 250 individuals were diagnosed with HIV-1 infection in Korea before 1993. Among the 250 individuals, we selected all who remain healthy for >18 years in the absence of antiretroviral therapy. They were four individuals (26, 25, 21, and 18 years from diagnosis). One patient who was diagnosed with HIV-1 in 1992 took a little KRG – less than 1,000 g. To investigate whether LTNPs are associated with genetic defects, we amplified near full-length HIV-1 sequences in 10 overlapping fragments (0.8 to 1.4 kb) using nested PCR with peripheral blood mononuclear cells. We compared the data with that in 4 control patients. Amount of KRG supplied was significantly higher in LTNPs (21,767-8,866 g) than 1,250-1,304 g in controls (P < 0.01).

## Results

We obtained 1,339 and 386 PCR amplicons over 20 years in LTNPs and over 10 years in controls, respectively. All LTNPs revealed at least one genetic defect in 4 genes, whereas controls revealed it in both 5’ LTR/*gag* and *nef* genes. Overall genetic defects were significantly higher in LTNPs (11.4%) than 4.9% in controls (P < 0.001). Among the 4 LTNPs, one patient who had taken the least amount of KRG revealed significantly higher proportion of premature stop codons (5.6%) than 1.6% in 3 LTNPs (P < 0.01). There was a significant difference in the frequency of genetic defects between on KRG (10.9%) and baseline prior to KRG intake (4.3%) (P < 0.01). There was a significant correlation between amount of KRG supplied and proportion of genetic defects (*r*=0.80, P < 0.05). At single gene level, only the *nef* gene showed a significantly higher frequency of genetic defects in LTNPs (11.5%) than 3.6% in controls (P < 0.01).

## Conclusion

These data show the possibility that frequent genetic defects that might be caused by KRG treatment are associated with long-term slow progression.

## References

[B1] Attele AS Wu JA Yuan CS Ginseng pharmacology: multiple constituents and multiple actions Biochem Pharmacol 1999 58 1685 93 10.1016/S0006-2952(99)00212-9 10571242

[B2] Sung H Kang SM Lee MS Kim TG Cho YK Korean red ginseng slows depletion of CD4T cells in HIV-1-infected patients Clin Diagn Lab Immunol 2005 12 497 501 15817756 10.1128/CDLI.12.4.497-501.2005 PMC1074393

